# Bare-Part Color in Female Budgerigars Changes from Brown to Structural Blue following Testosterone Treatment but Is Not Strongly Masculinized

**DOI:** 10.1371/journal.pone.0086849

**Published:** 2014-01-24

**Authors:** Stefanie E. P. Lahaye, Marcel Eens, Veerle M. Darras, Rianne Pinxten

**Affiliations:** 1 Research Group Ethology, University of Antwerp, Wilrijk, Belgium; 2 Laboratory of Comparative Endocrinology, KU Leuven, Leuven, Belgium; Arizona State University, United States of America

## Abstract

Whereas several studies have shown that experimentally increased levels of the androgenic steroid testosterone can affect female behavior, fewer studies have focused on the activational effects of exogenous testosterone on female morphology. With respect to colorful displays in birds, almost exclusively the effects of testosterone manipulation on female carotenoid-based colorations have been studied. Other color types such as structural colors (i.e. UV, blue and violet colors that result from differential light reflection in the nanostructures of the tissue) remain largely unstudied. Here, we investigated the short- and long-term effects of exogenous testosterone on the expression of structural bare-part coloration in female budgerigars, *Melopsittacus undulatus*. In this parrot species, bare-part coloration is expressed in the cere, a structure over the beak which is brown in females and structural blue in males. We experimentally increased plasma testosterone levels in testosterone-treated females (T-females) compared to controls (C-females) and we performed weekly spectrophotometric measurements of the cere for five weeks after implantation and one measurement after ten weeks. We also estimated the extent to which testosterone masculinized female cere color by comparing the experimental females with untreated males. We found significant effects of testosterone on cere color from week four after implantation onwards. T-females expressed significantly bluer ceres than C-females with higher values for brightness and UV reflectance. T-female cere color, however, remained significantly less blue than in males, while values for brightness and UV reflectance were significantly higher in T-females than in males. Our quantitative results show that exogenous testosterone induces the expression of structural blue color in females but does not strongly masculinize female cere coloration. We provide several potential pathways for the action of testosterone on structural color.

## Introduction

The effects of the androgenic hormone testosterone (T) on male traits such as ornamentation, courtship and mating behaviors have been well documented, particularly in birds (e.g. [1]–[3]). Although T is often regarded as a male hormone, a large body of correlational as well as experimental studies illustrates that T also acts in females (e.g. [4]–[6]). Moreover, implantation studies have shown that exogenous T can enhance the expression of male-typical traits in females of many species (reviewed in [4]). Several studies have addressed the activational effects of T with respect to behavioral traits, in particular song behavior, in females of a range of bird species (e.g. [7]–[10], see [4] for a review). In contrast, fewer studies have examined the effects of exogenous T on female morphological characteristics.

The bright color displays in male birds are among the most intensively studied morphological traits in the animal kingdom (e.g. [11], [12]). Especially the evolution of colorful plumage traits through sexual selection has received considerable attention [13], [14]. In contrast to plumage coloration, bare-part colors can change rapidly in response to the current physiological condition of its bearer [15]–[17]. Being dynamic, such colorations can be used for example to continuously estimate mate quality, before as well as after pair formation [17]. Accordingly, several studies indicate that females as well as males adjust reproductive investment after pairing based on bare-part coloration of their mate [18]–[22]. Whereas the hormonal control of male plumage coloration varies among species, the color of male bare-parts (e.g. eye rings, bills, legs and featherless regions of the face and neck) is generally under the control of T [1], [23]. Similarly as in males, it has been shown that bare-part coloration is sensitive to male-like levels of T in females of some species. For example T-implantation of female European starlings, *Sturnus vulgaris*, causes their bill color to change from black to yellow [9]. Zebra finches, *Taeniopygia guttata*, diamond doves, *Geopelia cuneata*, and moorhens, *Gallinula chloropus*, show red-orange bare-part coloration of respectively the bill, the periorbital ring and the frontal shield. Treating females with exogenous T results in the expression of brighter colors in these bare-part structures [24]–[26]. As bare-part coloration is a dynamic trait, such color changes are expected to take place within a short-term period [17]. Experimental studies have shown that bare-part color in males can change in response to a variety of cues, including increased T-levels, over short periods of two to four weeks (e.g. [27]–[29]) or even within one week (e.g. [16], [30], [31]). Studies investigating the effects of exogenous T in females have reported dynamic changes of bare-part coloration after two [9], [24] up to eight weeks [26]. However, in most studies the timing of T-induced color changes have not been monitored in detail (but see [9] for an exception).

Remarkably, all of the studies on T in females that are mentioned above investigated carotenoid-based yellow, orange or red colors. Although widespread among birds and other taxa, the hormonal control of other color types such as melanin-based and structural colors has been largely neglected ([23], but see: [32]). Structural coloration results from differential light reflection in the nanostructures of the tissue and includes UV, blue, violet and some green colors [33]. As far as we are aware, only two studies have addressed the effects of T on structural color in females and these studies have yielded conflicting results. T-manipulation of male superb fairy-wrens, *Malurus cyaneus*, in winter causes them to molt into their brightly colored blue nuptial plumage, suggesting that T is involved in the regulation of structural blue plumage coloration [34]. In females of the same species, T also induces molt but although their feathers have a male-like morphology, male-typical blue coloration is not produced [35]. With respect to bare-part coloration, implantation and castration experiments have shown that in male Eastern fence lizards, *Sceloporus undulatus*, T is responsible for the expression of blue skin color [36]. Females are normally not blue, but blue skin color is induced in females by implanting them with T [36].

In contrast to plumage color, it may be that similarly as in lizards, structural bare-part coloration is sensitive to T in females of avian species. Structural bare-part coloration is found in a variety of bird taxa [33]. The budgerigar, *Melopsittacus undulatus*, is a small nomadic member of the parrot family. In this species, bare-part coloration is expressed in the cere, a fleshy structure over the beak. Budgerigar cere color may function as a sexual signal as many courtship interactions are performed while the members of a pair are positioned face to face, putting the cere centrally in the visual field of the birds [37]. Moreover, both females and males show mating preferences based on cere coloration (e.g. [38], [39], but see: [40]). Cere color is sexually dimorphic. Males express bright blue structural color in the cere, while females cere color ranges from whitish-blue to dark brown [37]. Juvenile males express pink-purple cere color [37], [38]. During sexual maturation, the color changes to blue and this process is fastened by implanting juvenile males with T, which indicates that cere color is T-dependent in male budgerigars [37], [38]. In a study by Nespor et al. [41] on the effects of T on the vocal behavior of female budgerigars, it was mentioned anecdotally that implanting adult females with T causes cere coloration to change from brown to structural-based blue but quantitative data were lacking.

In this study, we quantitatively investigated the activational effects of exogenous T on the expression of structural bare-part coloration in females of the budgerigar. We manipulated plasma T levels in female budgerigars using implants filled with T (T-females) or left empty (C-females). We hypothesize that increased levels of T are associated with a change in cere color from brown to blue. To test this assumption, we performed weekly objective spectrophotometric measurements for five weeks following T implantation to monitor short-term changes in cere color. We also took one measurement ten weeks after implantation to study the effects on the longer term. Although female bare-part color can be affected by exogenous T in some species, it may still be distinguishable from male coloration (e.g. [26]). Hence, in addition we compared female cere coloration after implantation and natural blue male color to estimate the extent to which T causes a masculinization of female cere coloration.

## Materials and Methods

### Ethics Statement

The budgerigars used in this study were domesticated animals which were used to human presence. Because budgerigars are social birds, we always allowed the birds at least vocal interactions. We bled the birds to obtain approximately 300 µL blood from the alar vain, which represents less than 1% of the body weight and does not cause adverse effects [42]. The females behaved normally within a few minutes after blood sampling. We used Xylocaine (10% spray) to locally anaesthetize the females during the implantation procedure. Implantation did not cause more apparent distress than blood sampling. We found no immediate adverse effects after treatment or later on. Implantation (see ‘Hormone manipulations and blood sampling’) did not affect female survival and previous experiences have shown that T- and C-females reproduce successfully in subsequent breeding seasons (unpublished data). In addition, we did not observe abnormal behavior performed by the T- and C-females during the daily routine checks and no implant-related discomforts were observed. Handling time was minimized and did not exceed 3 min per individual for all procedures. All experimental procedures were performed in agreement with the Belgian and Flemish laws and were approved by the ethical committee of the University of Antwerp (ID number 2011/25).

### Study Species & Housing

We randomly selected 32 adult female budgerigars of approximately two years old from our captive stock. The birds had been obtained from local breeders as juveniles and had been maintained in our captive stock for two years. All females had a green plumage and showed cere coloration within the adult female range [37]. The birds had previously been housed in a single-sex outdoor aviary (8 m wide×2.5 m deep×2.3 m high). Two weeks before implantation (see ‘Hormone manipulations and blood sampling’), the females were housed individually in indoor cages (60 cm wide×40 cm deep×50 cm high) in one single room with auditory but no visual contact. The birds were maintained on a light regime of 15∶9 (L:D). Food (commercial budgerigar seed mix, Nifra Van Camp bvba, Belgium) and water were provided ad libitum.

### Hormone Manipulations and Blood Sampling

On 23 June 2011, females from both treatments received a silastic tubing implant (Degania silicone; length: 7 mm, 1.47 mm i.d., 1.96 mm o.d.). Implants were packed with 4 mm of crystalline T (Fluka 86500, 3.52±0.03 mg) or left empty and sealed on both ends with silastic glue. Implants of this size were selected based on previous work in female budgerigars because they successfully increase plasma blood levels to the male-like level within two weeks after implantation [43]. Captive male budgerigars show plasma T levels which are five to 20 times higher than in females and which vary from 0.5±0.1 ng/mL up to 1.2±1.0 ng/mL throughout the breeding season [38]. Implants were inserted subcutaneously along the left flank under local anesthesia (Xylocaine, 10% spray) and the incision was sealed with tissue adhesive (Histoacryl, Braun). Females were matched for body weight prior to assignment to either the testosterone group (T-females, n = 16) or the control group (C-females, n = 16). As we were particularly interested in the potential T-induced color changes of the cere, we additionally matched females for the initial variation in female cere color based on blue chroma calculated from reflectance spectrometric data (see ‘Spectrometry’). Blood samples were collected immediately before implantation and seven weeks after implantation. Blood samples were centrifuged at 7000 rpm for 10 min within two hours after sampling. The plasma fraction of 50–150 µL was removed and stored at −70°C until a hormone assay was performed. Plasma T concentrations were quantified by radioimmunoassay (RIA) using a commercial double antibody system purchased from MP Biomedicals (Solon, Ohio). Our hormone assay techniques have been reported previously [44]. Briefly, 500 µL of a 50/50 mixture of cyclohexane/ethylacetate was added to 50 µL plasma. After incubation, the tubes were extracted twice and the organic phase was transferred to a new tube and dried by vacuum centrifugation. The dried samples were dissolved in 25 µL steroid diluent buffer and further treated following the protocol of the RIA kit. The specification sheet provided by the company indicates that the primary antibody used in this assay does not cross-react significantly with other androgens beside T (5α-dihydrotestosterone: 3.4%; 5α-androstane-3β,17β-diol: 2.2%; 11-oxo-testosterone: 2%; all other steroids: <1%). T standards ranged from 0.1 ng/mL to 10 ng/mL but the effective detection limit could be extended to 0.05 ng/mL owing to the concentration effect of the extraction procedure. The intra-assay coefficient of variation was 4.6−9.1% (medium - low/high concentrations) and all samples were measured within the same assay.

### Spectrometry

The reflectance spectra of the cere of all females were measured with an USB4000 spectrophotometer (Ocean Optics, Duiven, The Netherlands), using an Ocean Optics DH-2000 BAL deuterium/halogen lamp. The spectral range between 320 and 700 nm was included because it corresponds with the limits of vision in birds [45]–[47]. Before each measurement session we took a dark current measurement on the cere of a randomly selected live bird and a white standard reference measurement (WS-1, Diffuse Reflectance Standard, Ocean Optics, Duiven, The Netherlands) for calibration purposes. Next, the left side of the cere of all individuals was measured three times by the same person. Cere color of the females was measured one week before implantation and six times while the females carried their implant (one, two, three, four, five and ten weeks after implantation). We refer to these measurements as week −1, 1, 2, 3, 4, 5 and 10. One T-female died between week 5 and week 10 so no measurement for week 10 could be included for this female. The cause of death remained inconclusive as the female did not show any implant related or other external injury and no abnormally low or high body weight.

The largest source of variation between reflectance spectra is represented by mean reflectance as a measure of “brightness” (often 80% or more of the variation; (e.g. [48]–[51]), whereas the major objective of many studies, including the present study, is to investigate differences in color which are characterized by the shape of the spectrum [52]. We decided to separate the analysis of mean reflectance and spectral shape at an early stage. This method was proposed by Endler [53] and has been applied by several studies (e.g. [51], [52], [54]). We standardized each spectrum by subtracting its brightness, calculated as mean reflectance (R_320–700 nm_; [55]), across all wavelengths [52]. This standardization was performed separately for each of the three reflectance spectra per measurement. The mean of these three standardized reflectance spectra was then used in further analyses.

To quantify color objectively, we performed principal components analyses (PCA) on the standardized reflectance data [52]. PCA is a method to reduce a large number of correlated variables into a few orthogonal variables, the principal components (PC), which summarize most of the variation and are independent of one another [52], [56]. Each reflectance spectrum (320 to 700 nm) comprises 1928 data points. These were first reduced to the means of 10-nm steps, resulting in 38 data points [51]. In a PCA on raw spectral data the first PC score would typically capture variation in brightness because, as stated before, often the majority of variation between spectra is represented by brightness [52]. By using the standardized reflectance data, we made sure that all PC scores represented variation in spectral shape, while the effects of treatment on brightness were investigated using mean reflectance which was separately calculated as a measure for brightness. To facilitate the interpretation of our PC scores, we calculated four commonly used colorimetric variables and compared these with our PC scores [51]. We calculated UV chroma, blue chroma, green chroma and red chroma respectively as the proportion of total reflectance occurring between 320–400 nm (R_320–400_/R_320–700_), 400–500 nm (R_400–500_/R_320–700_), 500–600 nm (R_500–600_/R_320–700_) and 600–700 nm (R_600–700_/R_320–700_) [55]. For each individual, we first calculated the colorimetrics separately for the three spectra that were measured and then we calculated the mean of these three values which was used in the statistical analyses [57].

To estimate the extent to which T induced male-like cere color in females, we compared cere color of the experimental females 10 weeks after implantation with male cere color. We used the measurements of week 10 because at that point cere color of the experimental females had not changed significantly for six weeks (see ‘Results’), suggesting that maximal T-induced color changes had been reached. We did not include males in our experimental design. Hence, for this comparison, we used measurements of 40 untreated adult males that had been taken in May as part of a different study measuring the mating preferences of females (Lahaye et al., in preparation). These males were not part of the experimental study but as the natural ranges of cere coloration of adult male and female budgerigars do not show any overlap year-round ([38]; our personal observations), we are convinced that this comparison provides reliable and relevant information. The males were all of the same age as the experimental females (approximately two years old) when they were measured and prior to the measurements they had been housed in single-sex cages. The color measurements of the males, the standardization of the reflectance spectra, the calculation of the PCA and the colorimetrics were done identically as described above.

### Interpretation of PC Scores

**Figure 1:**
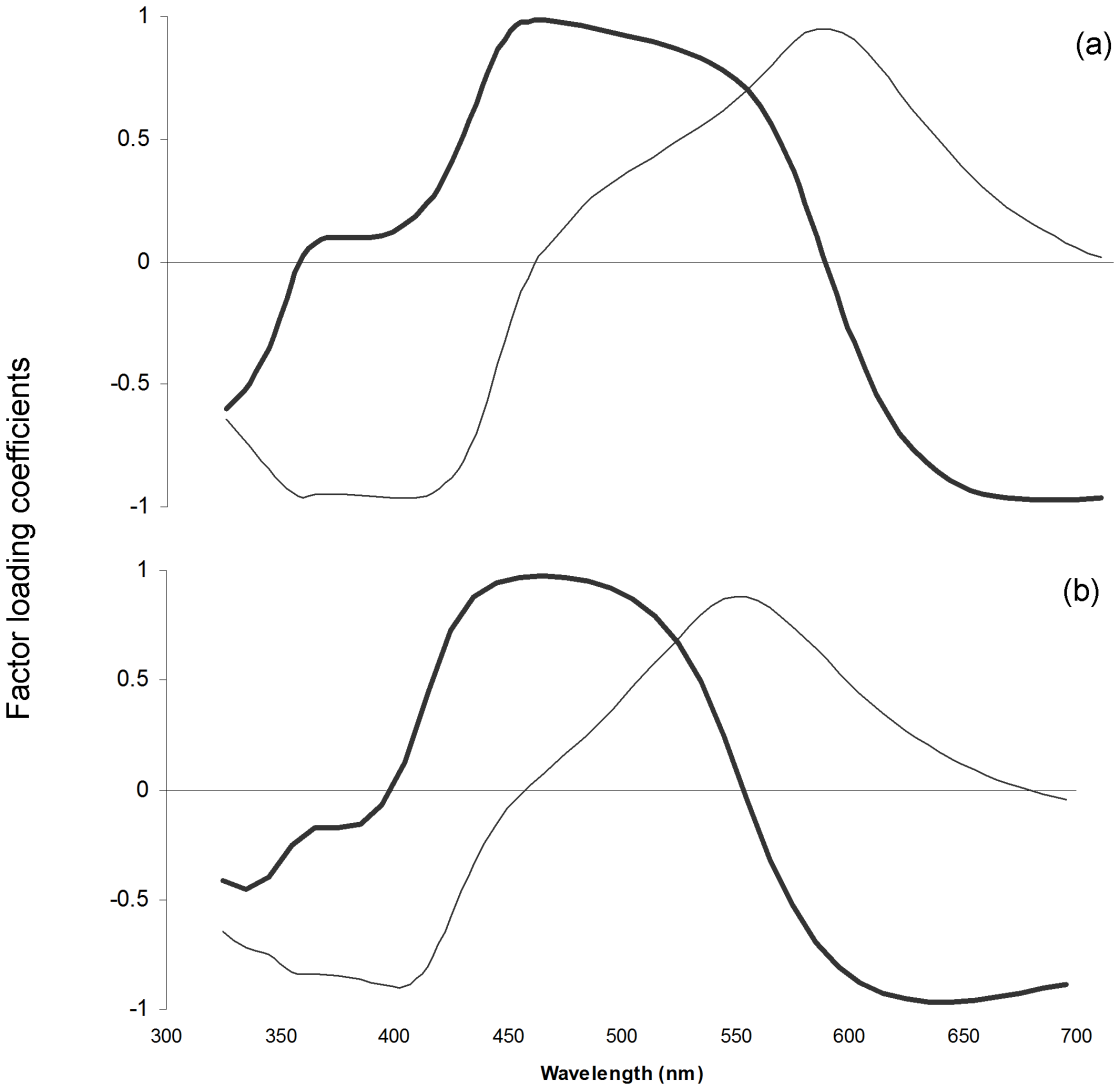
Association between principal component factor loading coefficients for PC1 (thick lines) and PC2 (thin lines). Coefficients are given for (a) C-females (n = 16) and T-females (n = 16; week_10_: n = 15) before implantation and one, two, three, four, five and ten weeks after implantation and (b) C- and T-females ten weeks after implantation (n_C_ = 16, n_T_ = 15) and untreated males (n = 40).

PCA including the spectral data of T- and C-females from week −1 until week 10 yielded two PCs that explained more than 90% of the variation in our dataset (PC1∶51.0%; PC2∶43.5%). The factor loadings for PC1 and PC2 are shown in figure 1a. The factor loadings for the PCs obtained through PCA including the spectral of untreated males and of T- and C-females 10 weeks after implantation are shown in figure 1b. Only the first two PCs were used, since together they explained more than 85% of the variation in our dataset (PC1∶52.6%; PC2∶34.0%). In both cases, PC1 was positively correlated with blue and negatively with red chroma and PC2 was negatively correlated with UV chroma and positively correlated with green chroma (table 1). PC1 was also correlated positively with green chroma for the PCA including the spectral data of T- and C-females from week −1 until week 10 and negatively with UV chroma for the PCA including the spectral data of untreated males and of T- and C-females 10 weeks after implantation (table 1). Altogether, these results suggest that PC1 represents the variance in intermediate wavelength reflectance (blue wavelength reflectance), whereas PC2 captured the variance in saturation, i.e. the relative reflectance in short (UV reflectance) versus medium wavelengths (green reflectance).

**Table 1:** Correlation coefficients between principal component scores (PC) and reflectance indices (see ‘Material & Methods’ for details).

	T - C				T - C - Male			
	PC1		PC2		PC1		PC2	
	r	*P*	r	*P*	r	*P*	r	*P*
UV chroma (320–400 nm)	0.12	0.09	−**0.81**	**<0.0001**	−**0.28**	**0.02**	−**0.51**	**<0.0001**
Blue chroma (400–500 nm)	**0.80**	**<0.0001**	−0.09	0.18	**0.71**	**<0.0001**	−0.21	0.08
Green chroma (500–600 nm)	**0.44**	**<0.0001**	**0.61**	**<0.0001**	0.08	0.49	**0.56**	**<0.0001**
Red chroma (600–700 nm)	−**0.75**	**<0.0001**	0.07	0.29	−**0.60**	**<0.0001**	0.23	0.06

Values in the columns with caption “T-C” and “T-C-Male” represent respectively correlations for the PCs obtained through PCA on the spectral data of cere color of T- and C-females for week −1 until week 10 and through PCA on the spectral data of cere color of untreated males and T- and C-females for week 10. Significant correlations are in bold (*P*<0.05).

### Statistical Analyses

We analyzed all data using the statistical package SAS® 9.2 (SAS Institute, Cary, NC, 2008). Data were checked for normality and, if not normally distributed, log (x+1) transformed. We performed Pearson’s correlations to compare colorimetric variables to PC scores. We used repeated measures ANOVA to investigate the effect of treatment (T or C) on plasma T levels and on cere color. Respectively plasma T concentration, brightness, PC1 and PC2 (obtained through PCA on the data of T- and C-females for week −1, 1, 2, 3, 4, 5 and 10) were included as dependent variables. Treatment, measurement (for plasma T concentration: before and after implantation; for brightness, PC1 and PC2: week −1 until week 10) and their interaction were included as explanatory fixed variables. In each model, female identity was included in the repeated statement. Because repeated observations were made on single individuals, residual values may be correlated. Therefore, we tested several covariance structures (i.e. compound symmetry, serial autocorrelation and unstructured) to select the best fitting regression model based on BIC values. The Satterthwaite correction was used to adjust the degrees of freedom [58]. We used one-way ANOVA to investigate the differences in cere coloration between untreated adult males and T- and C-females in week 10. Respectively brightness, PC1 and PC2 (obtained through PCA including the spectral data of the untreated males and of the T- and C-females) were included as dependent variables. We included bird category (male, T- and C-female) as fixed factor in the model. Within all SAS models these analyses were followed by post-hoc comparisons using t-statistics with adjusted p-values (p_a_) for multiple testing using Tukey corrections. Values are reported as mean ± SE. Significance was calculated at the *P*<0.05 significance level.

## Results

Before implantation, females from either experimental group did not differ in body weight (*t*_15_ = −0.32, *P* = 0.75) or in blue chroma of the cere (*t*_15_ = −0.90, *P* = 0.38), indicating that females were randomly distributed between both treatments.

### Plasma T Concentrations

Implantation significantly increased plasma T concentration in T-females but did not affect T concentrations of C-females (treatment*sampling interaction: *F*_1,26.7_ = 20.01, *P*<0.0001). Before manipulation, plasma T concentration did not differ between treatments (T-females: 0.27±0.10 ng/mL, C-females: 0.33±0.17 ng/mL; *t*_44.9_ = 0.23, *P*_a_>0.99) while seven weeks after implantation, T-females showed significantly higher plasma T levels than C-females (T-females: 2.05±0.30 ng/mL, C-females: 0.11±0.02 ng/mL; *t*_45_ = −5.77, *P*_a_<0.001).

### Effect of T on Cere Color

**Figure 2:**
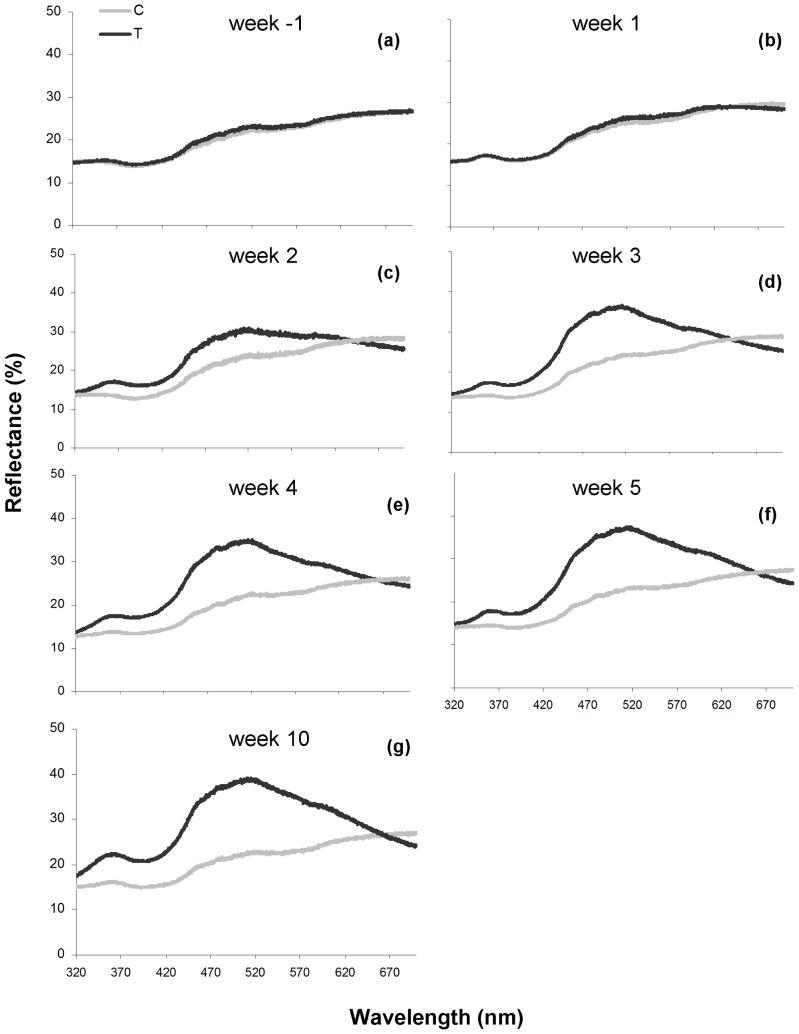
Cere color of T- and C-females. Reflectance spectrophotometry results for the cere color of C-females (n = 16, gray line) and T-females (n = 16; week_10_: n = 15, black line) before implantation (a), one (b), two (c), three (d), four (e), five (f) and ten (g) weeks after implantation.

**Figure 3:**
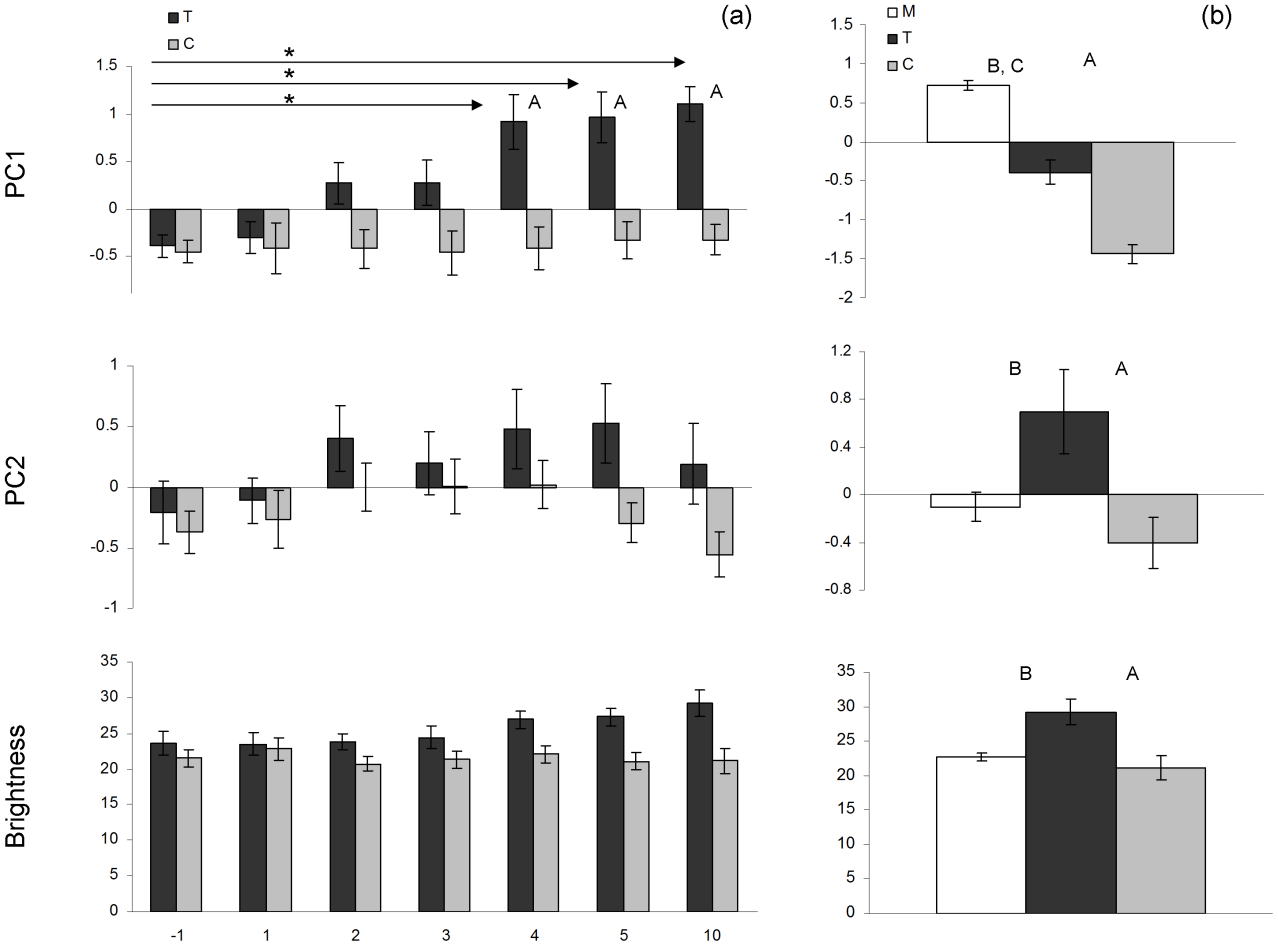
Mean ± SE principal component scores and brightness. PC1 represents blue wavelength reflectance and PC2 represents UV reflectance. Scores are given for (a) C-females (n = 16, gray bars) and T-females (n = 16; week_10_: n = 15, black bars) before implantation and one, two, three, four, five and ten weeks after implantation (asterisks (*) indicate significant differences between measurements for T-females, *P*<0.05) and (b) untreated males (n = 40, open bars) and T-females (n = 15, black bars) and C-females (n = 16, gray bars) ten weeks after implantation. (letters above histograms indicate significant differences between bird categories, *P*<0.05; A: difference between T- and C-female, B: difference between T-female and male, C: difference between C-female and male).

Measurements of the cere color of T- and C-females before implantation and 1, 2, 3, 4, 5 and 10 weeks after implantation are shown in figure 2. Reflectance spectra of the cere before implantation showed an increase across the spectrum (fig. 2a). T-treatment resulted in a spectrum with a peak in the blue part of the spectrum and a minor second peak in the UV part of the spectrum (fig. 2e–g). For blue wavelength reflectance (represented by PC1) we found a significant treatment*measurement interaction effect (*F*_6, 166_ = 3.40, *P* = 0.0034, fig. 3a). The effects of treatment and measurement were also significant (treatment: *F*_1, 43.5_ = 14.97, *P* = 0.0004; measurement: *F*_6, 166_ = 4.25, *P* = 0.0005). Post-hoc tests show that from week 4 onwards T-females showed significantly higher values for blue wavelength reflectance than C-females (table 2). C-females showed no significant differences for cere color before and after implantation (table 2). From week 4 onwards T-females showed significant higher values for blue wavelength reflectance than before implantation (table 2). Cere color of T-females measured in week 4, 5 and 10 did not differ significantly (table 2). For UV reflectance (represented by PC2) and brightness we could not find a significant treatment*measurement interaction effect (UV reflectance: *F*_6, 159_ = 0.80, *P* = 0.57; brightness: *F*_6, 144_ = 1.24, *P* = 0.29, fig. 3a, but see also ‘Male and female cere color’). There was a significant effect of measurement for UV reflectance (*F*_6, 165_ = 2.79, *P* = 0.013) but for treatment there was only a trend (F_1, 39.3_ = 3.12, *P* = 0.085). For brightness, the effect of measurement was not significant (*F*_6, 149_ = 1.18, *P* = 0.32) but there was a significant overall treatment effect (*F*_1, 40.9_ = 11.19, *P* = 0.0018).

**Table 2:** Post-hoc tests for the treatment*measurement interaction effect for blue wavelength reflectance of the cere.

	T			C		
	df	*t*	*P*	df	*t*	*P*
**Before-after**						
week-1–1	176	−0.56	>0.99	176	−0.21	>0.99
week-1–2	209	−2.89	0.19	206	0.03	>0.99
week-1–3	203	−3.26	0.08	203	−0.14	>0.99
week-1–4	198	−5.28	**<0.0001**	198	−0.13	>0.99
week-1–5	181	−5.22	**<0.0001**	181	−0.47	>0.99
week-1–10	165	−5.27	**<0.0001**	163	−0.47	>0.99
week4–5	176	−0.31	>0.99	176	−0.57	>0.99
week4–10	203	−0.55	>0.99	203	−0.45	>0.99
week5–10	178	−0.41	>0.99	176	−0.02	>0.99
**T versus C**						
week-1	100	−0.21	>0.99			
week1	100	−0.40	>0.99			
week2	100	−2.57	0.37			
week3	100	−2.43	0.47			
week4	100	−4.68	**0.0005**			
week5	100	−4.54	**0.0009**			
week10	100	−4.72	**0.0004**			

Post-hoc tests are given for the comparison between the measurements before (week −1) and after implantation (week 1–10) for respectively T-females and C-females and for the comparison between T-females and C-females for each measurement. Values in the columns with caption “T” and “C” represent the degrees of freedom, *t*-value and P-value for respectively T-females and C-females. Significant differences are in bold (*P*<0.05).

### Male and Female Cere Color

**Figure 4:**
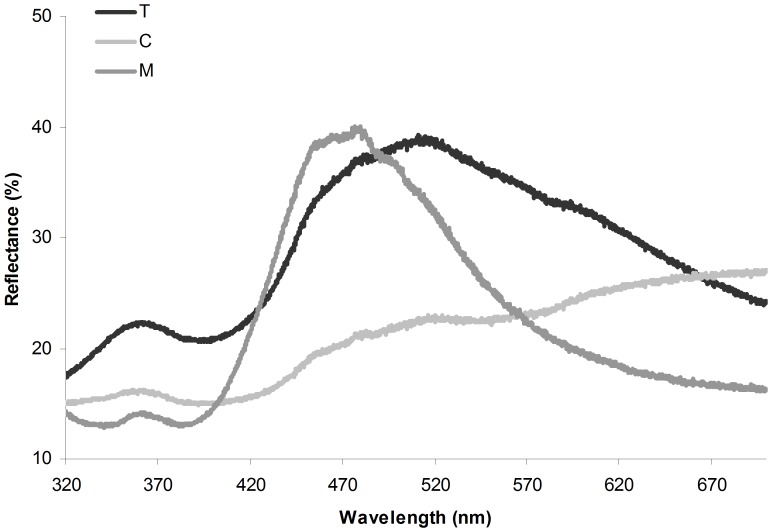
Female and male cere color. Reflectance spectrophotometry results from the cere color of C-females (n = 16, clear gray line) and T-females (n = 15, black line) ten weeks after implantation and untreated males (n = 40, dark grey line).

Measurements of the cere of untreated adult males and of T-and C-females in week 10 are shown in figure 4. For blue wavelength reflectance (represented by PC1) we found a significant difference between the three bird categories (*F*_2,68_ = 138.9, *P*<0.0001, fig. 3b). T-females as well as C-females showed significantly lower values for blue wavelength reflectance than males (T-females: *t*_68_ = 8.17, *P*_a_<0.0001; C-females: *t*_68_ = 16.23, *P*_a_<0.0001) and blue wavelength reflectance was significantly higher in T-females compared to C-females (*t*_68_ = 6.48, *P*_a_<0.0001). For UV reflectance (represented by PC2) we also found a difference between the three bird categories (*F*_2,68_ = 5.85, *P* = 0.0045, fig. 3b). C-females did not differ from males (*t*_68_ = −1.09, *P*_a_ = 0.53) but T-females showed significantly higher values for UV reflectance than males (*t*_68_ = −2.81, *P*_a_ = 0.018) and C-females (*t*_68_ = −3.26, *P*_a_ = 0.005). For brightness we found a significant effect of bird category as well (*F*_2,68_ = 10.44, *P* = 0.0001, fig. 3b). C-females and males did not differ (*t*_68_ = −1.03, *P*_a_ = 0.56), while T-females showed a significantly higher brightness compared to males (*t*_68_ = −3.99, *P*_a_ = 0.0005) and C-females (*t*_68_ = −4.21, *P*_a_ = 0.0002).

## Discussion

In this study we increased plasma T concentrations in T-females to the male-like level and we found that within four weeks after implantation T-females expressed significantly bluer coloration of the cere than C-females. In addition, we found that ten weeks after implantation cere coloration of T-females was still significantly less blue than in males.

### Effect of T on Cere Color

We found an effect of T-treatment on PC1 which represents blue wavelength reflectance of the cere relative to the rest of the reflectance spectrum. C-females showed no differences in blue wavelength reflectance for any of the color measurements before and after implantation. Blue wavelength reflectance of T-females before and one and two weeks after implantation did not differ significantly. Three weeks after implantation there was a trend for an effect of T on blue wavelength reflectance. From week four after implantation onwards, the values for blue wavelength reflectance differed strongly significantly from the values before implantation. Once a color change was detected, it remained fairly constant and there were no more significant changes in blue wavelength reflectance of T-females between weeks four and ten. Similar results were found when comparing T-females with C-females. There was no significant treatment*measurement interaction effect for UV reflectance, represented by PC2, or brightness, represented by mean reflectance. Possibly we could not detect a significant interaction for both traits because the color changes took place gradually. For brightness, we did find a significant overall treatment effect, while there was no significant effect of measurement. This supports that gradual color changes may have remained undetected because the elaborate dataset included several sampling points that had been taken shortly after implantation and for which the color changes may have been minor. For UV reflectance it may also be that the interaction effect was not significant because of the relatively large standard errors as we found only a trend for the overall treatment effect while the overall measurement effect was significant. Our results show that T treatment induces blue cere coloration which differs significantly from the coloration of controls after four weeks.

In contrast to previous studies investigating the hormonal control of bare-part coloration in females [9], [24]–[26], we did not focus on carotenoid-based yellow or red color but on structural-based coloration. As T-females expressed significantly bluer ceres, we can conclude that, similarly as carotenoid-based bare-part color, some structural-based colors seem to be sensitive to male-like levels of T in females. Furthermore, we found that T induces structural-based bare-part coloration with similar reaction times as described for T-induced changes in carotenoid-based coloration (e.g. [9]).

### Male and female Cere Color

Although T-females expressed significantly bluer ceres, cere color was not strongly masculinized. T-females expressed less blue ceres than males as values for PC1, which represent blue wavelength reflectance, were still significantly higher in males. In contrast, we found that T-females and males differed significantly for UV reflectance and brightness while C-females and males did not differ significantly for both traits. Hence, our results show that T-females are even less similar to males than C-females with respect to both traits. T-females showed significantly higher values for brightness than males. Interestingly, T-females also showed significantly higher values for UV reflectance, represented by PC2. T-induced effects on UV reflectance have been described for plumage and bare-part color in several species [24], [59], [60]. In budgerigars, as shown by the reflectance spectra of untreated males and females measured in this study, reflection in the UV part of the spectrum is normally low in both sexes (see also [61] for an additional spectrum of the male cere). In contrast, the cere usually shows a UV reflectance peak in raptor species [62], [63]. It is unclear why T caused T-females to show higher values for UV reflectance compared to males, which normally express low UV reflectance. Our findings indicate that T affected color in T-females, but did not induce male-typical blue color. Similarly, T-treatment of female zebra finches results in the expression of brighter carotenoid-based red bills, which are still less red than in males [26]. In other species, such as the diamond dove and the moorhen, the extent to which T affects carotenoid-based bare-part colors does not seem to differ between the sexes [24], [25]. As we found that T-females were even less similar to males than C-females for UV reflectance and brightness, our results indicate that in some species the expression of certain aspects of bare-part coloration may also be increased compared to males following T-treatment. Studying the effects of T in males and females of different species may help to increase our understanding of differential effects of this steroid hormone on traits in both sexes.

PCA including males, T-and C-females revealed a significant difference between T-and C-females, not only for blue wavelength reflectance (represented by PC1) but also for UV reflectance (represented by PC2) and brightness. T-females expressed significantly higher values than C-females for all three traits. These results suggest that T-treatment also has strongly significant effects on UV reflectance and brightness of the cere. However, PCA including spectral data of T-and C-females from before implantation and week 1–10 after implantation did not reveal similarly strong significant differences for both colorimetrics. This was especially not the case for UV reflectance as we found only a trend for the overall treatment effect. It is not clear why there is such a difference in results between both analyses. Possibly there is no complete overlap for the variance in saturation that is captured by the PC2 values we obtained with both PCA. Our results indicate that T seems to affect UV reflectance of bare-part color in females but more evidence to support this assumption is needed.

Our experimental results agree with previous qualitative observations on four individuals which suggested that in female budgerigar cere color is sensitive to T [41]. In the previous study it was reported that cere color changed to the blue, male-like condition within two weeks after implantation [41]. In contrast we only detected a significant T-induced effect on female cere color after four weeks and we found that cere coloration, even after 10 weeks, still differed significantly from the male condition. This discrepancy may be due to the fact that we used objective, spectrophotometric measurements to quantify color, supporting that this method is likely to provide more accurate results than assessment of color using human vision [52].

### Pathways for the Hormonal Control of Structural Coloration

Our results show that T-treatment affects structural coloration in female budgerigars but the mechanisms underlying this effect remain to be investigated. In male blue tits, *Cyanistes caeruleus*, T-treatment may influence UV reflectance of the crow feathers by stimulating grooming of the plumage [60]. We did not investigate whether T has a similar effect on grooming of the cere (by rubbing the cere against a perch or other object), but as we found marked effects of T which included a shift in coloration from brown to blue, it is less likely that our results can be explained by an effect on grooming behavior alone. Hence, it seems that physiological rather than behavioral mechanisms are responsible for the color changes observed in T-treated female budgerigars. Whereas recent studies have provided more insights into the hormonal control of carotenoid-based bare-part coloration (e.g. [23], [24], [26]), the pathways for the hormonal control of structural coloration are largely unstudied. We propose several non-mutually exclusive possibilities for our observations in female budgerigars.

Possibly, T influences the nanostructure of the skin of the cere, thereby promoting structural color. Correlational evidence suggests that T may affect the keratin structure of feathers [57]. As far as we are aware, it remains to be investigated experimentally whether T has the potential to influence structural coloration by causing changes of the nanostructure of tissues. In some cases colors are produced by a combination of structural color and pigmentation [33]. For example in the blue-footed booby, *Sula nebouxii*, blue foot color results from carotenoid pigmentation and the nanostructure of the skin [16]. If pigment-based coloration is sensitive to T, consequently combined colors may also change in response to T. Parrots, such as the budgerigar, do not express carotenoid-based coloration but color based on psittacofulvins, pigments unique to the parrot family [64]. It is not known whether these pigments are present in the cere of budgerigar and whether psittacofulvin-based coloration is sensitive to T, thus providing a mechanism for T regulation of combined structural colors. Alternatively, T may act on melanin pigmentation in the outer tissue layers of the cere. For example in males of the American Goldfinch, *Carduelis tristis*, T-manipulation induces the removal of melanin and in combination with carotenoid deposition, this results in the expression of colorful bills [65]. The cere color of female budgerigars is brown, most likely because of the presence of melanin [66], [67]. Similarly as in goldfinches, T may cause a withdrawal of melanin from the outer tissue layers of the cere, revealing the potential underlying blue structural color [38], [65]. Interestingly, female budgerigars develop darker brown cere color in response to estradiol (E_2_) treatment and treating females with an anti-estrogen causes the cere color to turn blue [38], [68], [69], indicating that estrogens may be important regulators of female brown cere color possibly through an effect on melanin deposition. These findings seem to suggest that female budgerigar cere color is sensitive to the action of both E_2_ and T. However, we have to point out that, although T-treatment caused changes of female cere coloration, it could be that our observations are not caused by T itself but by products of T metabolism such as E_2_ or 5α-dihydrotestosterone. To strictly being able to separate the effects of T and the products of T metabolism, it is necessary to prevent the conversion of T into these metabolites or to use the corresponding receptor blockers.

Clearly, more research is needed to elucidate the mechanisms through which T influences structural color and to estimate the potential role of T-metabolites in this process. When studying the pathways for the hormonal control of structural coloration, it may also be important to take into account the possibility that colors could be affected through different physiological mechanisms in males and females. Such gender-specific mechanisms have been described for the effects of T on carotenoid-based bare-part coloration in zebra finches [26]. In this species, T-treated females express less brightly colored beaks than males [26]. This sex-dependent effect of T may be due to the fact that T-females experience a decrease in body mass, indicating that T-females experience a metabolic cost, while in males no such effect has been found [26], [70]. As fat tissue is thought to be a sink for carotenoid pigments, females with lower body mass may have lower circulating carotenoid concentrations at their disposal and therefore less brightly colored beaks [26], [71]. Our results show that in some species T may act differently on structural color in both sexes as well. We found that exogenous T influences female cere coloration but does not cause the expression of bright blue color typical of males. Similarly, superb fairy-wrens fail to express male-typical blue plumage coloration following T-treatment [35]. We also found that the expression of brightness and UV reflectance of the cere is increased, even compared to males. The potential gender-specific mechanisms underlying the sex differences in color expression we found are unknown. Studying these mechanisms may help to increase our understanding of the hormonal control of sexual dimorphism. Within the parrot family, many species show little sexual dimorphism in coloration while in some species males express brighter coloration and in a few species it are even the females that are brighter colored (e.g. [67], [72], [73], [74]). Therefore, this taxon may provide a very interesting model system to further study sex-specific effect of T on structural-based as well as pigment-based colorations of bare-part and plumage traits.

To summarize, we found that experimentally elevating plasma T levels in female budgerigars to the male-like level affects bare-part coloration of the cere. T-females show higher values for brightness and UV reflectance than males. Nevertheless, the production of structural bright blue color seems to be limited to males because, although T-females express bluer ceres, cere color is not fully masculinized. Whether females are unable to develop male cere color due to organizational effects of sex steroid hormones early in ontogeny [75], [76] or whether cere color is controlled by other mechanisms such as non-hormonal direct genetic effects [77] remains to be investigated.
